# Silk fibroin nanoparticles dyeing indocyanine green for imaging-guided photo-thermal therapy of glioblastoma

**DOI:** 10.1080/10717544.2018.1428244

**Published:** 2018-01-25

**Authors:** He-Lin Xu, De-Li ZhuGe, Pian-Pian Chen, Meng-Qi Tong, Meng-Ting Lin, Xue Jiang, Ya-Wen Zheng, Bin Chen, Xiao-Kun Li, Ying-Zheng Zhao

**Affiliations:** a Department of Pharmaceutics, School of Pharmaceutical Sciences, Wenzhou Medical University, Wenzhou City, Zhejiang Province, China;; b Department of Ultrasonography, The First Affiliated Hospital of Wenzhou Medical University, Wenzhou City, Zhejiang Province, China

**Keywords:** Silk fibroin, nanoparticles, indocyanine green, phototherapy, glioblastoma

## Abstract

Silk was easily dyed in traditional textile industry because of its strong affinity to many colorants. Herein, the biocompatible silk fibroin was firstly extracted from *Bombyx mori* silkworm cocoons. And SF nanoparticles (SFNPs) were prepared for dyeing indocyanine green (ICG) and construct a therapeutic nano-platform (ICG-SFNPs) for photo-thermal therapy of glioblastoma. ICG was easily encapsulated into SFNPs with a very high encapsulation efficiency reaching to 97.7 ± 1.1%. ICG-SFNPs exhibited a spherical morphology with a mean particle size of 209.4 ± 1.4 nm and a negative zeta potential of −31.9 mV, exhibiting a good stability in physiological medium. Moreover, ICG-SFNPs showed a slow release profile of ICG *in vitro*, and only 24.51 ± 2.27% of the encapsulated ICG was released even at 72 h. Meanwhile, ICG-SFNPs exhibited a more stable photo-thermal effect than free ICG after exposure to near-infrared irradiation. The temperature of ICG-SFNPs rapidly increased by 33.9 °C within 10 min and maintained for a longer time. ICG-SFNPs were also easily internalized with C_6_ tumor cells *in vitro*, and a strong red fluorescence of ICG was observed in cytoplasm for cellular imaging. *In vivo* imaging showed that ICG-SFNPs were effectively accumulated inside tumor site of C_6_ glioma-bearing Xenograft nude mice through vein injection. Moreover, the temperature of tumor site was rapidly rising up to kill tumor cells after local NIR irradiation. After treatment, its growth was completely suppressed with the relative tumor volume of 0.55 ± 033 while free ICG of 33.72 ± 1.90. Overall, ICG-SFNPs may be an effective therapeutic means for intraoperative phototherapy and imaging.

## 1. Introduction

Glioblastoma, especially Glioblastoma multiforme (GBM), is far from defected by mankind. GBM was classified by world health organization (WHO) as IV grade, which was the most serious glioma types (Bush et al., 2017). The rate for patients with malignant gliomas achieved 3.19 cases in 100,000 per years, which constituted 45.2% of total central system malignant and people received surgery therapy finally disappointing with the high rates of recurrence and mortality within two years due to the invasive growth of malignant tumor and unclear defining border of tumor against normal cells (Bush et al., 2017; Wang et al., 2017). The current strategies treating with GBM expect to achieve more accurate positioning with selectively distinguishing tumor boundary and more thorough treatment combining with multiple drugs or clinical methods, like ultrasound (Lin et al., 2016), light (Guan et al., 2017), thermal (Feng et al., 2015), magnetic (Xu et al., 2016).

Phototherapy, represented by photodynamic therapy (PDT) and photo-thermal therapy, is a noninvasive and effective approach for cancer treatment. In phototherapy, light is administered, absorbed by the photosensitizer (PS) or photo-thermal agent, and converted into reactive oxygen species (ROS) or local hyperthermia, leading to tumor cell death. To selectively and efficiently destroy the targeted cancer cells while sparing normal tissue, sufficiently enhance the tumor accumulation of phototherapeutic agent, precisely focus the laser beam on the tumor areas, and adequately convert the absorbed light energy to ROS or heat are important. Fluorescence (FL) imaging in near-infrared (NIR) window (650–900 nm) holds much promise due to minimal autofluorescence and tissue scattering (Pansare et al., 2012). Many NIR dyes had been used for research such as cy5.5 (Veiseh et al., 2007), IR-780 (Ye et al., 2017) and indocyanine green (ICG) (Mundra et al., 2015; Sahu et al., 2016; Yan et al., 2016) for imaging and phototherapy of tumor. For example, ICG not only could real-time detect the tumor and its margin with normal tissue using contrast enhancement and spectrum-resolved technology, but also could absorb near-infrared light and produce a local thermal effect on tumor (Ma et al., 2013; Zhao et al., 2014; Beziere et al., 2015). However, the poor stability and short half-lives in blood limited its broad clinical application. For example, ICG was liable to aggregate and degrade in the aqueous solution, which result in its rapid clearance from liver (Pansare et al., 2012).

Recently, various delivery systems such as liposomes, dendrimers, and nanoparticles (NPs) have been developed for systemic PDT therapy. Systemic delivery of drug conjugated NPs can improve the bioavailability, stability, half-life, and clearance of drugs from the body. Various biomaterials, whether natural or synthetic, can be used for preparing NPs based drug delivery systems. A poly(lactide-co-glycolide) (PLGA) nanoparticle, which was modified by brain-targeting peptide, angiopep-2, was prepared to load ICG together with docetaxel (DTX) for chemo- and phototherapy of glioma under near-infrared irradiation (Hao et al., 2015). In recent years, natural proteins based NPs have been more interested in biomedical fields due to their suitable biocompatibility, biodegradability, less immunogenicity, and the ability to well-tolerate with biological systems. For example, human serum albumin was a family of globular and water-soluble proteins with low immunogenicity and no toxicity. Human serum albumin (HSA)-ICG nanoparticles (HSA-ICG NPs) were prepared by intermolecular disulfide conjugations for dual-modal imaging-guided cancer synergistic phototherapy (Sheng et al., 2014). HSA-ICG NPs had a high accumulation with tumor-to-normal tissue ratio of 36.12 ± 5.12 at 24 h and a long-term retention with more than seven days in 4T1 tumor-bearing mice.

Alternatively, silk fibroin (SF) is a natural protein, which is usually originated from *Bombyx mori silkworm cocoons* used in textile industry for centuries. SF possesses a large molecular weight of 200–350 kDa or more and bulky, repetitive, modular hydrophobic domains of GAGAGS, which are interrupted by small hydrophilic groups. Apart from the primary structure, the secondary organization of SF dominates its gelatin property. SF possesses many superior properties including strong mechanical strength, flexible preparation process, biocompatibility and biodegradation for tissue engineering. SF has been used in a variety of biomedical applications and in different formats; specifically, it has been widely employed as medical sutures, for bone and cartilage tissue engineering or recently in clinical trials for the reconstruction of the tympanic membrane and breast implants. Although this biomaterial has broadly been used in local application in clinical or preclinical modes, few of them has been reported for intravenous application (Mottaghitalab et al., 2015). Recently, SF was prepared by the typical salting-out method and used as materials-preparing nanoparticles for intravenous delivery of doxorubicin, showing some superiority such as a good biocompatibility and a prolonging circulating time as PEGlyated liposome. SF nanoparticles were prepared to entrapment doxorubicin for targeting MCF-7/ADR tumor with the help of external magnet (Tian et al., 2014). Results indicated that the SF nanoparticles can prolong the doxorubicin blood circulation time and enhance efficient anti-tumor activity.

Silk fibroin was easily dyed in Chinese traditional textile industry because of its strong affinity to many colorants. The side chains of the amino acidic residues of SF can interact with pigments. For example, phenol red dye was easily entrapped within the β-sheet structures of drying silk films for pH sensing *in vitro* cell culture. Besides, pigment like chlorophyl α,β-carotene, and astaxanthin adsorbed onto SF are more stable to visible and UV light than fibroin-free pigments, which are rapidly decolorized by UV light irradiation. Moreover, a representative fluorescent dyes rhodamine B was used to feed silkworms and form dimers in body, which was easily absorbed into SF *in vivo* to trace xenobiotics in silkworm’s gland. These interesting phenomenon indicated a close interaction between the SF and certain natural dyes (Tansil et al., 2011).

The preparation of SF nanoparticles (SFNPs) is mostly based on a salting-out effect as described previously in the literature. It was usually difficult for this method to produce SFNP with the uniform size. Moreover, the resulting nanoparticles were susceptible to aggregate into non-dispersible clusters in the salting out process. Furthermore, the SF nanoparticles tend to aggregate in the biological medium like saline or PBS (10 mM, pH =7.3), which hinder the *in vivo* application of nanoparticles. It is usually required for SFNPs to be coated with the positively charged substance such as polyethylenimine (Wang et al., 2015), chitosan (Wang et al., 2015; Collado-González et al., 2016), or doxorubicin (Seib et al., 2013) to improve their stability in the medium solution. However, the cationic coating agents were usually toxic against the living body which brought SF nanoparticles intravenous administration into the deadlock.

**Figure 1.**
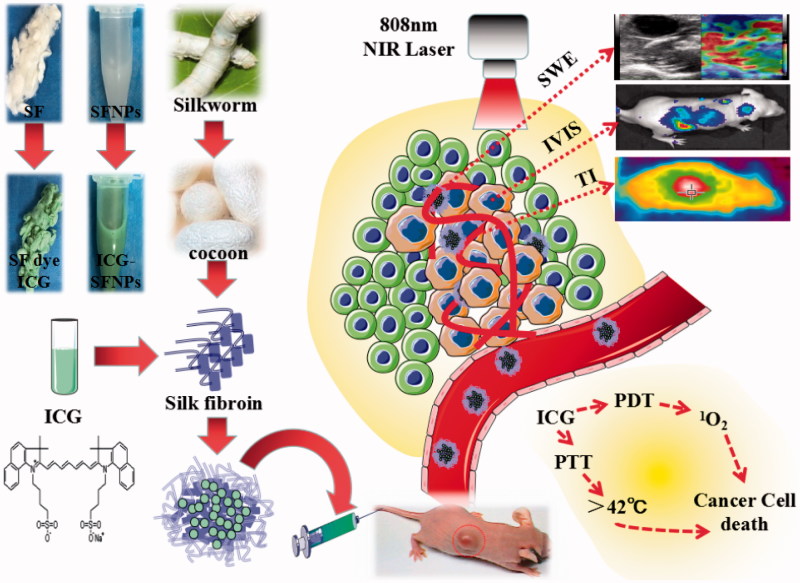
Schematic design of ICG-SFNPs and their potential clinical application for imaging and phototherapy of glioblastoma.

In this study, SF without immunogenicity was firstly extracted from the *B. mori silkworm* cocoons and SFNPs were prepared by an acetone-precipitation method. To produce a stable SFNP, the preparation of SF nanoparticles was carefully investigated by controlling the concentration of SF solution. ICG was encapsulated into SFNPs for imaging and phototherapy of glioblastoma (As illustrated in Figure 1). Meanwhile, the physiochemical properties of ICG-SFNPs including particle size, zeta potential and morphology and photo-thermal effect were also carefully characterized *in vitro*. Second, *in vitro* cell uptake and photo-induced cytotoxicity of ICG-SFNPs was also carefully studied by using by C_6_ cells line *in vitro*. Finally, *in vivo* distribution of ICG-SFNPs in C_6_ glioma-bearing Xenograft nude mice was performed by the *in vivo* imaging after intravenous injection. Meanwhile, the photo-thermal effect of ICG-SFNPs on tumor tissue was investigated by thermal imaging and temperature detector probe, and the growth inhibition of tumor was also monitored after intravenous administration of ICG-SFNPs followed by laser irradiation at 808 nm NIR. Finally, the stiffening of tumor after treatment was detected by ultrasound shear wave elastography (SWE).

## 2. Materials and methods

### 2.1. Materials

*Bombyx mori* Cocoons were collected from the Zhejiang huzhou Shuanglin huajie silk cotton opening factory. Na_2_CO_3_ and lithium bromide was purchased from Sigma-Aldrich Company (St Louis, MO, USA). Phosphotungstic acid and ICG were obtained from Meilunbio^®^ (Dianlian, China). Dialysis bag (3500 Da) was provided by Solarbio^®^ (Beijing, China). Dulbecco’s modified Eagle’s medium (DMEM), and fetal calf serum, and trypsin was provided by Gibco BRL^®^ (Grand Island, NY, USA). C_6_ cell line was obtained from the Institute of Biochemistry and Cell Biology, Shanghai Institutes for Biological Sciences, Chinese Academy of Sciences (Shanghai, China). Cell counting kit-8 (CCK-8) was purchased from Dojindo (Minato-ku, Japan).

### 2.2. Extraction of SF solution

Silk fibroin solution was prepared as the method previously described (Kim et al., 2016). Briefly, *B. mori* cocoons were cut into pieces and immersed in Na_2_CO_3_ solution (0.02 M). In order to remove sericin, the solution were heated and boiled for 1 h at 100 °C. Afterward, the boiled cocoons was withdrawn and rinsed with distilled water. After dying, the degummed fibers (10 g) were slowly dissolved in 200 mL of LiBr solution (9.3 M) at 60 °C for 3 h. And then, the flocculent precipitation was removed by the centrifugation at 5000 rpm and the supernatant was further filtrated to remove the tiny undissolved substance. Finally, the dissolved silk solution was dialyzed with dialysis bag (MWCO 3500) against 2000 mL of deionized water for 3 days to remove the LiBr, obtaining the dilute SF solution. The concentration of the resulting dilute SF solution was approximately 2 wt%, which was calculated by weighting the residual dry SF after fan dying of 10 ml SF solution. The dilute SF solution was further concentrated in vacuum drying oven at 65 °C and 4% of SF solution was finally obtained. The concentrated SF solution was stored in refrigerator at 4 °C for further use. During the degummed process, the residue sericin on the surface of silk fiber was monitored by the scanning electron microscope (H-7500, Hitachi, Japan). Finally, the molecular weight of the extracted SF was analyzed by SDS-PAGE.

### 2.3. Preparation and characterization of SFNPs andICG-dyed SF nanoparticles (ICG-SFNPs)

The SF nanoparticles were prepared as the method previous described (Hassani et al., 2017) with some modification. Briefly, to induce the desolvation process, SF solution (9 mL) was added dropwise (50 μl/drop) to acetone under vigorous stirring at room temperature. After addition of SF solution, the reaction solution became milk-white status and the result solution was further stirred overnight so that SF nanoparticles were completely dehydrated. Then the resulting SFNPs solution was further rotated to remove acetone and the remaining SFNPs were finally reconstituted into the distilled water followed by sonication at 300 W amplitude for 20 min (pulse of 1 s ON and 2 s OFF). Afterward, the SFNPs were filtered through a filter membrane (JinTeng, Tianjin, China) with 0.45 μm to remove contaminants. The filtered nanoparticle suspension was stored at 4 °C for further use. In order to screen the stable SFNPs, the different concentration of SF solution was used to prepare series of SFNPs for stability study. In order to ICG-dyed SFNPs, 1 mL of ICG solution (1 mg/ml) was firstly added to SF solution (9 mL) followed by the similar procedure as the preparation of SFNPs. Just, the final aqueous ICG-SFNPs solution was further added into ultrafiltration device (Millipore, Billerica, MA, USA) to remove the undyed ICG.

After the removal of the undyed ICG, the ICG-SFNPs was disrupted by adding 10% of sodium dodecyl sulfate (SDS) to release the dyed ICG in the nanoparticles (Mundra et al., 2015). The concentration of ICG was detected by UV–Vis spectrometer at 785 nm. The encapsulation efficiency (EE %) and the drug-loading capability (DL%) of ICG-SFNP was calculated by the following formula: EE (%) = (the weight of the dyed ICG)/(the weight of the theoretical ICG in formulation) × 100%; DL (%) = (the weight of dyed ICG)/(the total weight of NPs) × 100%.

### 2.4. Characterization of SFNPs and ICG-SFNPs

The hydraulic diameter (*D*_h_), polydispersity index (PDI) and zeta potential of SFNPs or ICG-SFNPs were detected by Zetasizer Nano ZS (Malvern, UK). The morphology of these nanoparticles was also assessed by Transmission Electron Microscopy (JEOLJEM-2000EX, JEOL, Japan) at 200 kV. Briefly, 10 μl of the nanoparticles solution was dropped onto a carbon-coated grid followed by staining with 2% phosphotungstic acid.

Fourier transform infra-red spectra (FT-IR) of the lyophilized SF powder, SFNPs and ICG-SFNPs were analyzed by FT-IR 8400 spectrophotometer (Shimadu, Japan) using the potassium bromide disk. The crystallinity of SFNPs were analyzed by powder X-ray diffraction (XRD) measurement (Rigaku RINT 2000; Rigaku Co., Tokyo, Japan) using CuΚα radiation with the scanning region of 10–50°. Differential scanning calorimetry (DSC) was performed on SFNPs to examine their thermal characteristics. The thermal change of the SFNPs powder was analyzed using differential scanning calorimeter (METTLER TOLEDO DSC822e, Zurich, Switzerland). SFNPs were scanned at room temperature and heated up to 400 °C at the rate of 20 °C/min.

### 2.5. Spectral properties of ICG-SFNPs

The UV–Vis (TU-1901, Beijing) spectrum of free ICG solution (20 μg/ml) was firstly detected by UV/Vis spectrometer from 400 to 850 nm. To confirm the successful encapsulation of ICG, the UV–Vis spectrum of ICG-SFNPs was also detected. Meanwhile, 10% SDS was added to ICG-SFNPs and incubated for 15 min to destroy its nanoparticles and release ICG. The spectrum of the released ICG was also recorded.

The spectral stability of ICG-SFNPs was measured by UV/Vis spectrometer. Briefly, ICG-SFNPs were added to pH7.4 PBS (10 mM, 150 mM NaCl) and further incubated at 37 °C for different time (the equivalent concentration of ICG in ICG-SFNPs was 20 μg/mL). The spectra of ICG-SFNPs were detected by UV–Vis.

### 2.6. The photo-thermal effect of ICG-SFNPs

In order to measure the photo-thermal effect of ICG-SFNPs, 2 mL of ICG-SFNPs (the corresponding concentration of ICG: 100 μg/ml) was added into the quartz curvette (Xinli, Jiangsu) and the temperature detector was contact with the solution. Using 1 W/cm^2^ laser to irradiate the sample for 40 min, respectively, the temperature changes of each sample were recorded by temperature detector probe (RC-4 Mini Temperature Data Logger; Elitech, Jiangsu, China) every 0.5 min. The real-time thermal imaging was recorded by thermal imaging imager (DT-9868, CEM). The PBS and free ICG were also measured as control.

In order to measure the photo-thermal circle stability of ICG-SFNPs, the temperature changing of ICG-SFNPs were measured by light on/off (5 min/15 min) for three times. The temperature were recorded every 0.5 min using temperature detector probe.

### 2.7. *In vitro* release of ICG-SFNPs

*In vitro* release profile of ICG from ICG-SFNPs was studied by dynamic dialysis method as depicted previously in literature (Tansil et al., 2011). Briefly, ICG-SFNPs (1 mL) were added into the dialysis bags and then the dialysis bags were submerged into 9 ml of pH7.4PBS containing 0.5% of tween-80 under the continuous shaking at 120 rpm at 37 °C. At different time interval, aliquots supernatant were taken out and the ICG concentration was measured by UV/Vis spectrometer as described above.

### 2.8. *In vitro* cell experiments

#### 2.8.1. Cell culture

C_6_ glioma cells were cultured in DMEM culture medium containing 10% fetal bovine serum, 1% penicillin and 1% streptomycin at 37 °C under a circumstance containing 5% CO_2_.

#### 2.8.2. *In vitro* cell uptake

C_6_ cells were seeded into six-well chambered coverglasses at the density of 5 × 10^4^ in 1 ml of culture medium. After 24 h culturing at 37 °C under circumstance containing 5% CO_2_, the medium was replaced by new medium with ICG-SFNPs or free ICG (30 μg/ml ICG). After 1, 4, 8 h incubation, the cells were rinsed with PBS (0.01 M, pH =7.4) for three times, fixed with 4% paraformaldehyde and finally, the cells were analyzed under confocal laser microscopy.

#### 2.8.3. *In vitro* cytotoxicity of ICG-SFNPs

Seeded C_6_ cells (5 × 10^3^/well) into the 96-well in 100 μl of medium. The plates were kept at 37 °C for 24 h to ensure that cells adhere to the bottom of walls. Then the medium was replaced by new medium containing ICG-SFNPs and free ICG. After incubation for 4 h, the cells were washed with PBS and incubation with medium. Each well was exposed to near-infrared light (1 W/cm^2^, 808 nm) for 3 min separately. The cells were incubated for 2 h under the CO_2_ circumstance and the cell viability was evaluated using CCK-8 assay.

### 2.9. *In vivo* study of the ICG-SFNPs

#### 2.9.1. Glioma BalB/C nude mice model

Male BalB/C nude mice (six to eight weeks) were purchased from Shanghai, China. All animal experiments were performed under the approval and guidance of the Institutional Animal Care and Use Committee of Wenzhou Medical University (SPF level). Briefly, C_6_ cells at the density of 1 × 10^7^ were subcutaneous injected in the right flanks of each mice.

#### 2.9.2. *In vivo* photo-thermal therapy

The ultrasound shear wave elastography (SWE) Images were acquired with the ultrasound device Aixplorer (SuperSonic Imagine; Aix-en-Provence, France) using a 15-MHz superficial probe dedicated to research (256 elements, 0.125 µm pitch). When the tumor reached 100 mm^3^, the mice were inhaled with 5% isoflurane gas and their body temperature was maintained at physiological level using a heating plate. The mice were injected with PBS, free ICG, ICG-SFNPs intravenously and the tumor site was facing up. The tumors were covered with a layer of standard ultrasound gel before measurement. The SWE scanning pictures of tumor site were acquired before and after 5 min irradiation.

When the tumor reached 100 mm^3^, tumor bearing mice was injected with ICG-SFNPs (200 μg/ml ICG). 808 nm NIR laser irradiation (1 W/cm^2^) was applied at tumor site for 5 min. The tumor sizes were measured by a caliper every day and calculated as the tumor volume = (tumor length) × (tumor width)^2^/2. Relative tumor volumes were calculated as *V*/*V*_0_ (*V*_0_ is the tumor volume when the treatment was initiated). The body weight of mice was measured every other day within the two weeks. Thermal imaging was conducted by near-infrared thermal imager before and after irradiation.

### 2.10. *In vivo* imaging and tumor accumulation effect

When the tumor reached 100 mm^3^, the *in vivo* FL images were taken by IVIS (IVIS^®^ Lumina II) after intravenous injected with ICG-SFNPs and free ICG (both 200 μg/ml). Filter set (Excitation Filter: 710 nm. Emission filter: ICG) was used for detecting ICG. After 12 h, tumor bearing rats were sacrificed. Main organs (heart, liver, spleen, lung and kidneys) and tumors were collected for determining the distribution of ICG-SFNPs and free ICG *ex vivo*.

### 2.11. Systemic toxicity

After mice were sacrificed, samples of heart, liver, spleen, lung and kidney tissue were collected and routinely stained with hematoxylin and eosin (H&E).

### 2.12. Statistical analysis

The level of significance in all statistical analyses was set at a probability of *p* < .05. Data are presented as mean ± SD. Analysis of variance and t-tests were used to analyze the data.

## 3. Results and discussions

### 3.1. Preparation and characterization of blank SFNPs/ICG-SFNPs

#### 3.1.1. Preparation of blank SFNPs/ICG-SFNPs

In this work, SFNP was prepared by organic solvent-induced self-assembly of SF chain. The noncovalent interactions (e.g. hydrogen bonding, hydrophobic effect) in SF chain in solution diminish and the disulfide bonds of albumin are cleaved, leading to protein unfolding into a linear structure such as a random coil/helix. Following addition to organic solvents such as ethanol, DMSO or acetone, the conformation of SF chain transited from a random coil/helix to β-sheet structures, which dominated self-assembly of SF into nanoparticles. This method eliminated the need for cross-linking agents and energy consumption (e.g. emulsification process by high-pressure homogenization), which was usually exploited to prepare protein-based nanoparticles.

Series of SFNPs were prepared by changing the SF concentration during self-assembly. Their *D*_h_ and PDI were detected by DLS and results were shown in Figure S2A. The nanoparticle size and polydispersity of SFNP were usually variable depending on SF concentration. As the concentration of SF increased from 0.5% to 2%, *D*_h_ of SFNPs gradually increased from 121.87 ± 3.57 nm to 237.8 ± 25.6 nm while PDI decreased accordingly from 0.838 ± 0.11 to 0.431 ± 0.02. Interestingly, when the concentration of SF was reaching to be 4%, *D*_h_ of SFNPs was 193.3 ± 5.43 nm and the particle distribution was very narrow with PDI of 0.103 ± 0.02. Inversely, when SF concentration further increased to be 8%, *D*_h_ and PDI of SFNPs increased again to be 352.3 ± 44.61 nm and 0.453 ± 0.01, respectively. The SF concentration-dependent *D*_h_ and PDI for SFNPs may be attributed to the different stability of these nanoparticles. In previous report, SFNPs was of instability after dispersing in PBS, DMEM or other biological media. Single nanoparticle was susceptible to aggregate or disassemble in these mediums, which resulted in the rapid enlargement of their particles size and broader particle size distribution (Wang et al., 2015). The stability of these SFNPs in pH7.4 PBS (10 mM, 150 mM NaCl) was monitored by DLS at different time interval and results were exhibited in Figure S2B. Truly, the particles size of SFNPs prepared by using 0.5% or 1% SF increased rapidly after adding SFNPs in the PBS solution. Only at 5 min, the particles size grew to 1437.17 ± 405.55 nm and 1525 ± 181.81 nm for these two types, respectively. When the concentration of SF increased to be 2%, the stability of SFNPs was slightly improved. After 5 min of incubation in the PBS, the particles size of SFNPs increased to 662.83 ± 48.84 and gradually aggregated with time. By contrast, as for 4% SF, the SFNPs were still stable, *D*_h_ maintaining at the level of ca.200 nm during the all tested time point. However, SFNPs prepared by 8% SF solution aggregated in PBS, *D*_h_ increasing from 321.1 ± 2.84 nm to 2596.33 ± 230.48 nm after 120 min of incubation. Alternatively, Tyndall effect of SFNPs in PBS solution was also real-time recorded by digital camera after ordinary red laser radiation (Figure S3). Consistent with the variation trends in particles size of SF-NPs, the aggregated particles for SFNPs by SF solution of 0.5% or 1% or 8% could be observed under naked eyes. For SFNPs prepared by 2% or 4%, the scattering light along light path was strong and uniform without any visible aggregates. But the reaction time in acetone has an important effect on the stability of SFNPs prepared by 2% SF solution in PBS.

The formation and stability of SFNPs was highly associated with the transition of secondary configuration of SF, as illustrated in Figure S2C. When SF was exposed into acetone, the secondary structure of GAGAGS block in SF molecule immediately changed from α-helix to β-sheet, forming hydrophobic segment. The hydrophobic chains from intra/inter-molecules were firstly dehydrated and formed hydrophobic core while the hydrophilic block between GAGAGS chains aggregated spontaneously to form outer shell of SFNPs. SFNPs prepared by low concentration of SF (0.5%, 1% and 2%) tended to float up because of the loose aggregation between GAGAGS chains, while SFNPs by a high concentration of SF (8%) showed tended to sediment because of its high density of SF inside nanoparticle. Only the SFNPs prepared by 4% of SF were stable in PBS, which has suitable particle size and polydispersity.

#### 3.1.2. Morphology and particle size of blank SFNPs/ICG-SFNPs

**Figure 2.**
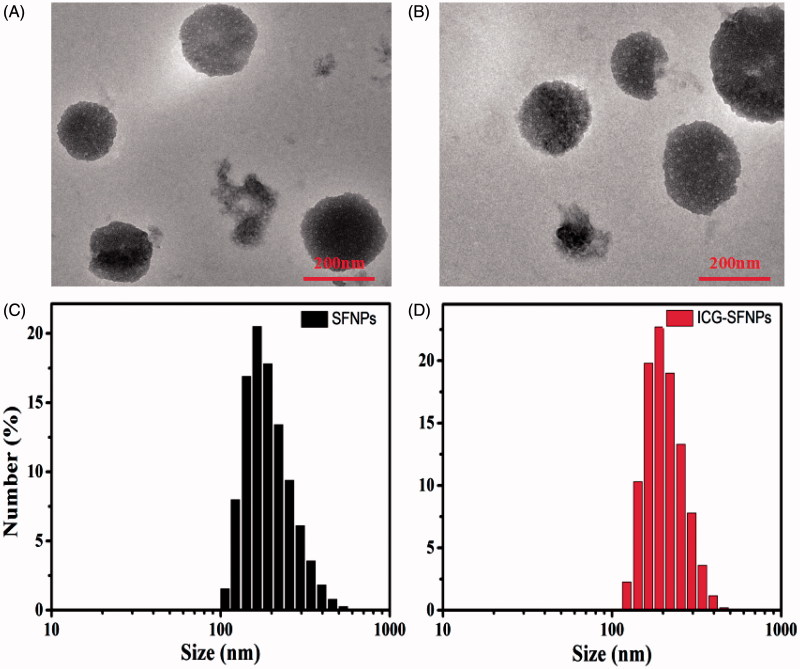
TEM images and particle size distribution of SFNPs (A, C) and ICG-SFNPs (B, D).

The morphology of SF-NPs and ICG-SFNPs were observed by TEM. TEM graphics of SF-NPs and ICG-SFNPs prepared by controlling SF concentration at 4% were shown in Figure 2(A, B). The sphere-like particles with the diameter of ca. 220 nm was visible regardless of encapsulating ICG or not in these graphics, indicating encapsulation of ICG did not compromise the self-assembly of SF chain. Moreover, the dynamic diameter and the size distribution of SFNPs and ICG-SFNPs were also measured by DLS. As shown in Figure 2(C, D), *D*_h_ and PDI of SFNPs was 198.9 nm and 0.1, while D_h_ of ICG-SFNPs was slightly increased to 209.4 nm and PDI was decreased to 0.04. Meanwhile, the surface zeta potential of SFNPs and ICG-SFNPs, were detected to be −35.6 and −31.9, respectively, which favored the stability of these SFNPs in the solution. The encapsulating efficiency of ICG in SFNPs was reaching to 97.7 ± 1.1%, indicating strong affinity of SF materials to ICG dye.

##### 3.1.3. The secondary structure transition of SF in blank SFNPs/ICG-SFNPs

**Figure 3.**
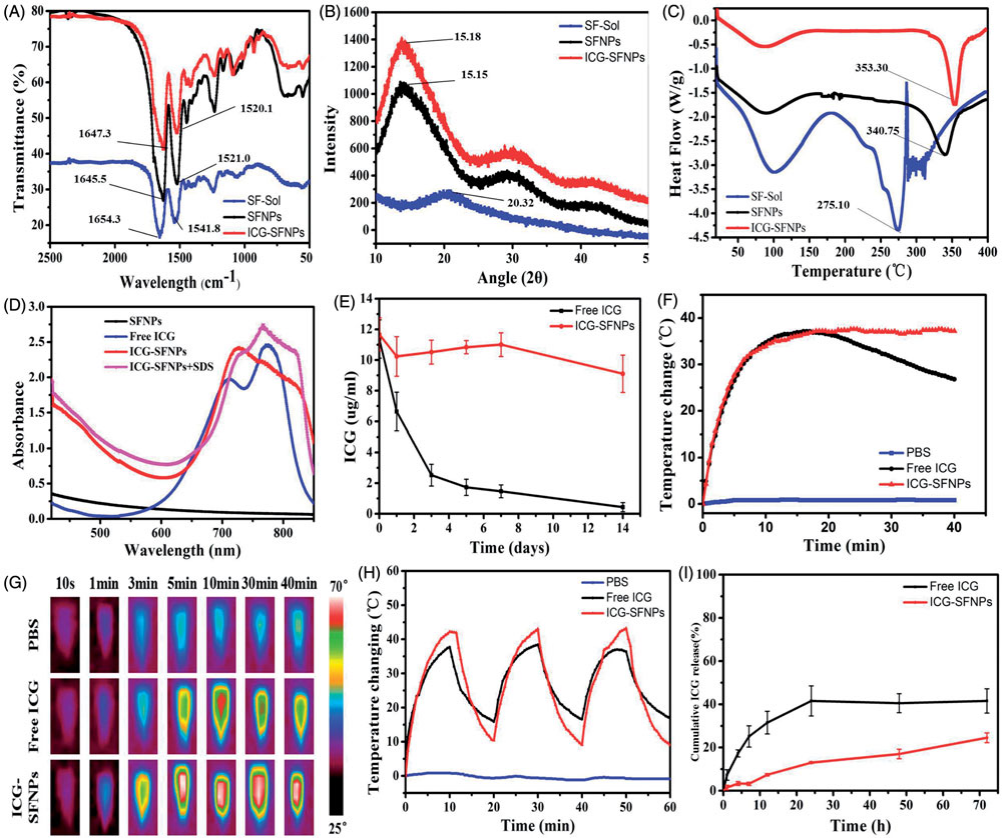
(A) FT-IR, (B) XRD and (C) DSC of the lyophilized SF powder, SFNPs, ICG-SFNPs, (D) UV–Vis peak absorbance of ICG in different formulations, (E) *In vitro* stability of ICG in various formulations after incubation with pH7.4 PBS for different time at 37 °C, and (F) the photo-thermal effect of ICG-SFNPs after exposure of laser light (808 nm NIR, 1 W/cm^2^ radiation) within 40 min, and (G) thermal imaging of various ICG-containing formulations after 10 min of laser radiation, (H) the photo-thermal stability of various ICG-containing formulations, and (I) *in vitro* release profiles of ICG from various ICG-containing formulations.

To confirm whether the secondary structure of SF transited from α-helix or random coil to β-sheet in nanoparticles, the lyophilized powder of SFNPs/ICG-SFNPs was investigated by FT-IR, XRD and DSC. As shown in Figure 3(A), the peaks at 1654.3 cm^−1^ and 1541.8 cm^−1^ attributed to amide I band and amide II band for the extracted SF solution shifted to 1645.5 cm^−1^ and 1521 cm^−1^ for SFNPs, respectively. These results were caused by the transition of the secondary structure of SF from random coil to β-sheet. Moreover, the transition was not compromised by encapsulation of ICG in SFNPs, in which the similar peaks shift occurred. The amorphous dispersion of the extracted SF also transited to the crystal form in the self-assembled SFNPs, which was reflected by results of XRD. There was merely crystal peak except a weak peak at 22.5° in XRD pattern of the extracted SF, suggesting the disordered arrangement of SF molecule. By contrast, a group of crystal peaks at 15.4°, 28.5° and 42.5° appeared in XRD pattern of the lyophilized SFNPs or ICG-SFNPs (Figure 3(B)). The crystal transformation of SF molecules further confirmed the transition of the secondary configuration of SF from α-helix or random coil to β-sheet. Likewise, the vast difference of heat behavior between the extracted SF and the SFNPs was also observed in DSC results. The melting point at 275.1 °C for the extracted SF powder was significantly lower than that of the SFNPs powder (340.7 °C) (Figure 3(C)), exhibiting its low crystal or amorphous status. Interestingly, the encapsulated ICG made the melting peak of SFNPs appearing at a higher temperature of 353.3 °C, indicating a strong interaction between ICG and SFNPs.

### 3.2. Spectral properties and photo-thermal effect ofICG-SFNPs *in vitro*

The UV–Vis spectrum of ICG-SFNPs was firstly investigated and results were shown in Figure 3(D). The blank SFNPs exhibited no absorption peaks at wavelength range of 500–800 nm. There existed two characteristic absorption peaks at 700 nm and 775 nm for free ICG solution. But one peaks of ICG shifted from 700 nm to 726 nm and another peak at 775 nm disappeared for ICG-SFNPs, indicating encapsulation of ICG inside SFNPs instead of the physical absorption on its surface. Moreover, when the detergent, SDS, was added to destroy the nanoparticle structure of ICG-SFNPs and release the encapsulated ICG (Li et al., 2017), two characteristic absorption peaks of ICG at 726 nm and 767 nm reappeared, suggesting the stable optical properties of ICG-SFNPs. However, it was reported that free ICG in the solution was susceptible to quench self-fluorescence property (Manchanda et al., 2010). Thus, the encapsulating stability of ICG in SFNPs was further evaluated by detecting the encapsulated ICG based on the absorption at 775 nm after addition of SDS. As shown in Figure 3(E), the encapsulated ICG was stable enough during two weeks test while free ICG solution rapidly decreased.

The photo-thermal effect of ICG-SFNPs was also evaluated and results were shown in Figure 3(F–H). The effect of the continuous NIR radiation (1 W/cm^2^) on temperature of PBS medium was negligible. By contrast, free ICG solution after continuous exposure of NIR radiation showed the rising temperatures and the elevating temperature was up to 42 °C after 10 min of radiation, at which the best anti-tumor effect was usually achieved for thermal therapy in multiple reports (Zhao et al., 2014; Beziere et al., 2015). The photo-thermal effect was also observed for ICG-SFNPs under NIR irradiation. Moreover, ICG-SFNPs exhibited a comparable extent in the rising temperature with free ICG solution, but temperature at platform stage for ICG-SFNPs remained for longer time under the continuous NIR irradiation while the platform temperature of free ICG solution gradually decreased after 20 min of NIR irradiation (Figure 3(F)). During NIR irradiation, the thermal picture of all the groups had been also real-time recorded by thermal imaging and result were shown in Figure 3(G). Compared with free ICG solution, the higher temperature and longer maintaining time were also observed for ICG-SFNPs. The photo-thermal effect of ICG-SFNPs under the discontinuous irradiation mode (ON = 10 min, OFF = 10 min, three cycles) was further investigated and the similar results was achieved (Figure 3(H)). Overall, these results suggested that the encapsulation of ICG in SFNPs might prevent its interaction with the surrounding environment and delay its decomposition thus improving the photo-thermal effect of ICG (Sheng et al., 2014).

### 3.3. *In vitro* drug release

*In vitro* drug release profile of ICG from SFNPs was studied in pH7.4 PBS containing 0.5% Tween-80 at 37 °C. As shown in Figure 3(I), free ICG rapidly diffused out from the dialysis bag within the 24 h and the release percentage reached a platform height, approximately 40% of the total ICG. The low platform height may be due to the decomposition of free ICG in the aqueous solution, which was confirmed by the results of stability in Figure 3(E). In contract, the encapsulated ICG was slowly released from SFNPs without any platform and the cumulative release percentage was only 24.51 ± 2.27% of the encapsulated ICG within 72 h. These results suggested a good stability of the encapsulated ICG in SFNPs and strong affinity between ICG and SFNPs to retain ICG (Salis et al., 2015). Because the strong physical interaction exist between ICG and SF, a slow release profile of ICG from SFNPs were exhibited, which was very helpful for them to target the tumor tissue through EPR effect (Enhanced Permeating Retention effect). Actually, the photo-thermal effect of ICG was not dependent on the release profile of ICG from SFNPs and the encapsulated ICG also exhibited a stable photo-thermal effect as shown in Figure 3(F–H). It was more advantageous for ICG-SF nanoparticles to stabilize ICG through the simple physical interaction, compared with the chemical crosslinking strategies in most of references (Nishi et al., 1995; Vandelli et al., 2001). Firstly, the self-assembly of SF was induced by organic solvent to encapsulate ICG without addition of any chemical crosslinking agent as reported in literature (Nishi et al., 1995). Secondly, the structure of the encapsulated ICG in SFNPs was not easily compromised, which was essential for them to practice the photo-thermal effect. But it was the most common for chemical crosslinking strategy to destroy the ICG structure and compromise its physiochemical properties.

### 3.4. *In vitro* cell uptake and cytotoxicity of ICG-SFNPs

**Figure 4.**
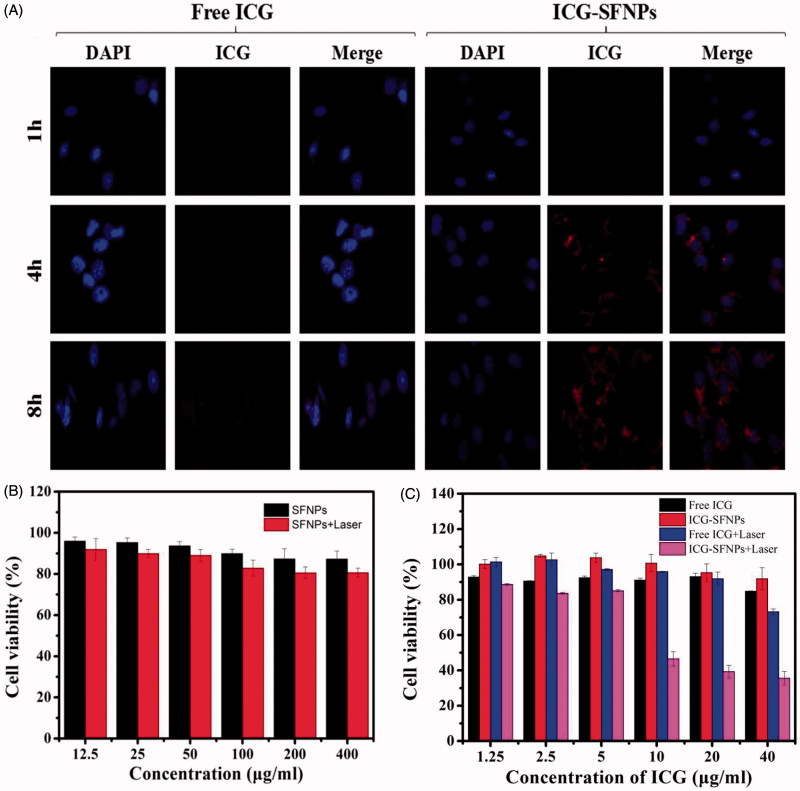
C_6_ Cell uptake and cytotoxicity of ICG-SFNPs: (A) confocal microscopy images of C_6_ after incubation with free ICG solution(30 μg/ml) and ICG-SFNPs(30 μg/ml equivalent ICG), and cytotoxicity of blank SFNPs (B) and ICG formulations (C) against C_6_ cells lines in presence or absence of irradiation.

The cellular uptake of free ICG and ICG-SFNPs by C_6_ cell line after 1, 4 and 8 h incubation was observed through confocal microscopy and results were exhibited in Figure 4(A). The time-dependent uptake of ICG-SFNPs was displayed, while there was not significant cellular uptake of free ICG solution. After 4 h of incubation, obvious red ICG FL was localized in cytoplasm of C_6_ cells treated with ICG-SFNPs and the FL intensity become more obvious as time increasing to 8 h. But there was not obvious FL of ICG in C_6_ cells treated with free ICG solution even at 8 h of incubation. The enhanced cellular uptake of ICG-SFNPs may due to the nanoparticle-specific endocytosis and improvement of stability for ICG encapsulated in SFNPs.

The cytotoxicity of ICG-SFNPs against C_6_ cells was further studied by CCK-8 assay. As shown in Figure 4(B), any cytotoxicity of SFNPs was not observed, more than 80% of cell viability, regardless of presence of NIR irradiation or not. Moreover, there was also not any toxicity of two ICG formulations including free ICG solution and ICG-SFNPs against C_6_ cells in absence of NIR irradiation. However, a dose-dependent cytotoxicity for ICG-SFNPs and free ICG solution was observed in presence of NIR irradiation. Moreover, the cytotoxicity of ICG-SFNPs was more obvious than that of free ICG solution at each test concentration. The trend in cytotoxicity of two ICG formulations was in accordance with intracellular ICG distribution as shown in Figure 4(A). These suggested that the thermal therapeutic effect might be dependent on intracellular concentration of ICG instead of its extracellular one.

### 3.5. *In vivo* distribution of ICG-SFNPs by *in vivo* imaging

**Figure 5.**
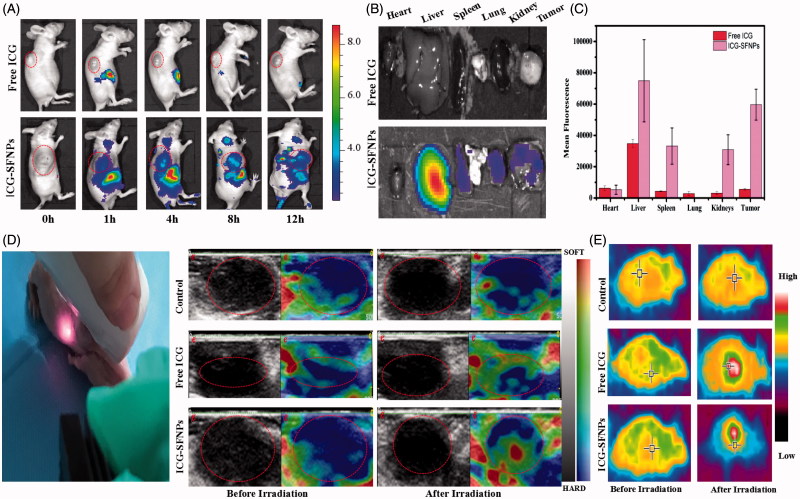
(A) *In vivo* imaging of BalB/C nude mice bearing C_6_ tumor cells at different time intervals after i.v. injection of free ICG solution or ICG-SFNPs, (B) *ex vivo* fluorescent images of major organs and tumors at 12 h after intravenous ICG formulations, (C) the statistical mean fluorescence intensity in *ex vivo* tissue, (D) *in vivo* ultrasound shear wave elastography (SWE) imaging of tumor (zone marked by the red circles) after 1 min of NIR irradiation, and (E) the thermal imaging of tumor bearing mice after 5 min of exposure to NIR laser (808 nm, 1 W/cm^2^).

C_6_ glioma-bearing Xenograft nude mice were constructed. When the size of tumor reached 100 mm^3^, the mice were intravenously injected with free ICG solution or ICG-SFNPs and *in vivo* imaging was acquired at different time point. As shown in Figure 5(A), free ICG was rapidly distributed to liver of mice, where a strong FL signal was displayed. As time increased, the ICG FL decreased gradually *in vivo* and disappeared completely at 12 h after administration. Meanwhile, there was merely any FL in tumor zone at each time point, indicating the poor distribution of ICG in tumor. On the contrary, after administration with ICG-SFNPs, besides liver, tumor tissues also some FL signal and its intensity became strengthened with time. At 8 h after intravenous injection of ICG-SFNPs, the FL inside tumor zone was strongest, and then became weaker with time (Figure 5(A)). Truly, the similar trend in FL was also observed by *ex vivo* fluorescent images of each organ and tumors at 12 h (Figure 5(B)). The FL intensity in tumor for ICG-SFNPs group was about eight times higher than that of free ICG solution (Figure 5(C)). The enhanced distribution of ICG in tumor for ICG-SFNPs may be due to the continuous accumulation of nanoparticles through enhanced permeating and retention effect (EPR effect) and the slower clearance of ICG-SFNPs (Gobin et al., 2006).

### 3.6. *In vivo* photo-thermal transition of ICG-SFNPs

To further determine the photo-thermal transition in tumor zone after administration of ICG-SFNPs, *in vivo* ultrasound shear wave elastography (SWE) was used to evaluate the tumor stiffening. Because the raising temperature will enhance the local blood circulation and make tissue become softer, SWE was commonly used to detect the photo-thermal effect of PS under irradiation (Marangon et al., 2017). At 8 h after intravenous injection of various formulations, the tumor-bearing mice were irradiated for 5 min by NIR laser and then SWE of tumor zone was immediately acquired. As shown in Figure 5(D), the mice treated with PBS showed no significant change of the tumor stiffening. Tumor stiffening was slightly changed in the groups treated with free ICG solution. By contrary, the tumor tissue of mice treated with ICG-SFNPs became obviously softer after irradiation, indicating the existence of photo-thermal transition. Alternatively, the temperature inside tumor zone was also detected by thermal imaging after NIR irradiation. As shown in the Figure 5(E), after administration of ICG-SFNPs, the temperature inside tumor zone significantly raised, exhibiting the better photo-thermal effect than free ICG solution. The effective photo-thermal transition was very helpful to produce thermal therapeutic effect on tumor.

### 3.7. *In vivo* inhibition *of* tumor growth

**Figure 6.**
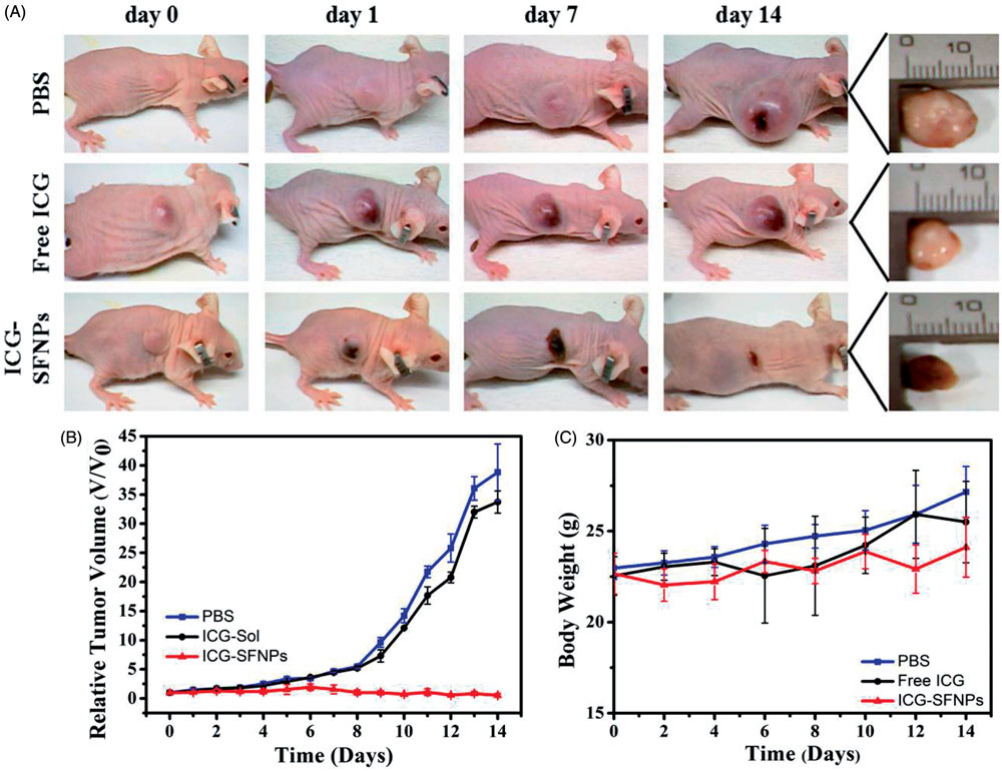
*In vivo* anti-tumor effect of ICG-SFNPs under irradiation, (A) photographs of C_6_ tumor-bearing mice at different interval after treatment with different formulations, (B) The calculated relative tumor size (*V*/*V*_0_) and (C) the body weight of the tumor-bearing mice at various stages.

*In vivo* anti-tumor efficacy of normal saline (Control), free ICG solution (DOX-S) and ICG-SFNPs was further evaluated in C_6_ tumor-bearing mice under NIR irradiation. As shown in Figure 6(A, B), tumors treated with saline grew faster than that in other groups. Although treatment with free ICG solution produced an inhibitory effect on growth of tumor at some extent, there existed no significant difference in tumor size between PBS group and free ICG solution group. The relative tumor volumes at 14 days after treatment with PBS + NIR and free ICG + NIR were 38.82 ± 4.87 (*p* < .005) and 33.72 ± 1.90 (*p* < .005), respectively. In contrast, the obvious inhibition of tumor growth was observed after treatment with ICG-SFNPs + NIR, and the relative tumor volume in this group decreased to 0.55 ± 0.33. Additionally, the body weight of tumor-bearing mice in all the groups grew slightly during treatment process, suggesting the low toxicity of these therapeutic means (Figure 6(C)). The inhibition of tumor growth was highly associated with temperature rising inside tumor zone. In PBS group, NIR irradiation without ICG was invalid to raise the temperature inside tumor zone and thus no inhibitory effect was observed in control group. Compared with treatment with free ICG solution, the higher accumulation of ICG-SFNPs inside tumor tissues raised significantly the temperature inside tumor zone after NIR irradiation, and therefore leaded to necrosis of the bulk tumor.

### 3.8. The systemic toxicity of ICG-SFNPs

The normal organs including heart, liver, spleen, lung and kidney was further analyzed by H&E staining to evaluate the toxicity of ICG-SFNPs. The results were shown in Figure S4. Regardless of treatment with free ICG solution or ICG-SFNPs, there was not any noticeable organ damage or inflammatory lesions in the heart, liver, spleen, lung or kidney of mice, indicating that ICG-SFNPs did not cause significant toxicity in the treated mice. Moreover, *in vitro* hemolysis study also confirmed ICG-SFNPs exhibited a good compatibility with blood (Figure S5). Overall, the ICG-SFNPs were safe to suit for intravenous systemic administration.

## 4. Conclusions

In summary, we used novel SF nanoparticle dyeing with ICG to construct a biocompatibility nano-platform (ICG-SFNPs) for photo-thermal therapy of C_6_ glioma. ICG was easily encapsulated into SFNPs with high encapsulation efficiency. ICG-SFNPs exhibited spherical morphology and negative zeta potential. Compared with ICG solution, ICG-SFNPs exhibited a more stable photo-thermal effect and slower release profile. Meanwhile, ICG-SFNPs were easily internalized with C_6_ tumor cells *in vitro*. Under the near-infrared irradiation, ICG-SFNPs showed a stable photo-thermal effect *in vitro*. *In vivo* imaging showed that ICG-SFNPs were effectively accumulated inside tumor site of C_6_ glioma-bearing Xenograft nude mice through vein injection. Finally, during two weeks treatment, the tumor growth was suppressed significantly compared with other control groups. Overall, ICG-SFNPs may be an effective therapeutic means for intraoperative phototherapy and imaging.

## Supplementary Material

IDRD_Xu_et_al_Supplemental_Content.docx
