# Supplementary material for: Silk fibroin nanoparticles dyeing indocyanine green for imaging-guided photo-thermal therapy of glioblastoma
Source: Drug Deliv. 2018 Jan 25;25(1):364–75. doi: 10.1080/10717544.2018.1428244 (PMC6058481; doi:10.1080/10717544.2018.1428244)
Supplement: IDRD_Xu_et_al_Supplemental_Content.docx [file IDRD_A_1428244_SM6821.docx]

**Supporting information**

**Extraction** **of silk fibroin solution**

The silk fiber of cocoon consists of 70% of fibroin inside center of silk fiber and 30% of sericin (a glue-like protein) on its outer surface. It was demonstrated that sericin could cause the severe allergic reactions when it was directly exposed to body. Silk fibroin was extracted from *Bombyx mori* cocoons according to the previously reported work. The extracting procedure of silk fibroin was shown in Fig S1A. The sericin on the surface of silk fiber were firstly removed by boiling the cocoon in 0.02% (w/v) Na_2_CO_3_ solution, which was called as “degumming process”. The surface of silk fiber was usually observed by scanning electron microscope (SEM) to confirm the removal status of sericin after/before degumming of silk fiber. Results were exhibited in Fig S1 B&C. There was an obvious curl membrane of sericin wrapping on surface of silk fibroin before degumming of silk fiber, making the surface of silk fiber be rough and untidy (Fig S1 B). By contrast, the surface of silk fiber became very smooth and neat (Fig S1 C) after degumming in 0.02% (w/v) Na_2_CO_3_ solution at 100°C for 1h, suggesting the complete removal of sericin. This was further demonstrated by the weight change of silk fiber at different time interval during degumming (Fig S1 D). Compared with its original weight, the weight loss of the boiled silk fiber reached the platform value of 31.2% after 1h of degumming, which was comparable to the practical content of sericin (30%) in the silk fiber of cocoon in most of publications^1^.

The degummed silk was water-insoluble because the silk fibroin existed in form of β-sheet structures which are orderly aligned along the axis of the silk fibers. The chains are held together by strong interactions, discovered to be hydrogen bonds between amide linkages of the adjacent chains. The hydrogen bond interaction inside silk fibroin fiber was destroyed in 9.3M LiBr solution, which resulted in the transition of SF conformation from β-sheet in silk fiber to a random coil/helix in the solution. The molecular weight of the dissolved SF was further detected by SDS-PAGE analysis. Two broad bands at molecular weight of ca.31 KDa and ca.238 KDa were dominantly observed for the extracted SF solution. Besides, the bands at molecular weight of approximately 300KDa and 25KDa were also detected in extracted SF sample, which was attributed to the heavy and light chain of silk fibroin in the literature^2^, respectively (Fig S 1E).


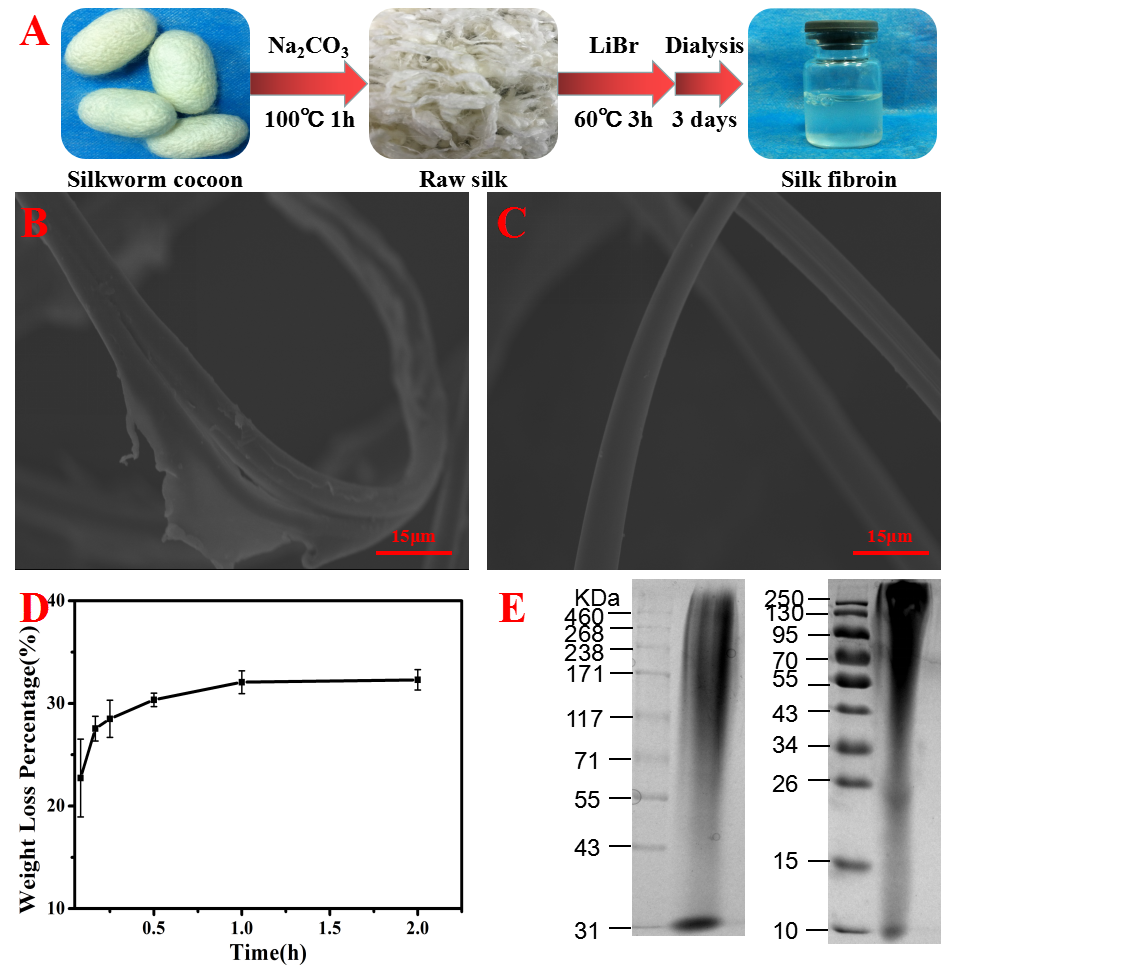


Fig S1 Extraction and characterization of silk fibroin. (A) Processing of silk fibroin extraction. Scanning electron microscope (SEM) for (B) raw silk and (C) silk after degumming. (D) Weight ratio of degumming silk to raw silk. (E) SDS-PAGE analysis of silk fibroin solution, right: high molecular weight marker and protein band, left: low molecular weight marker and protein band.


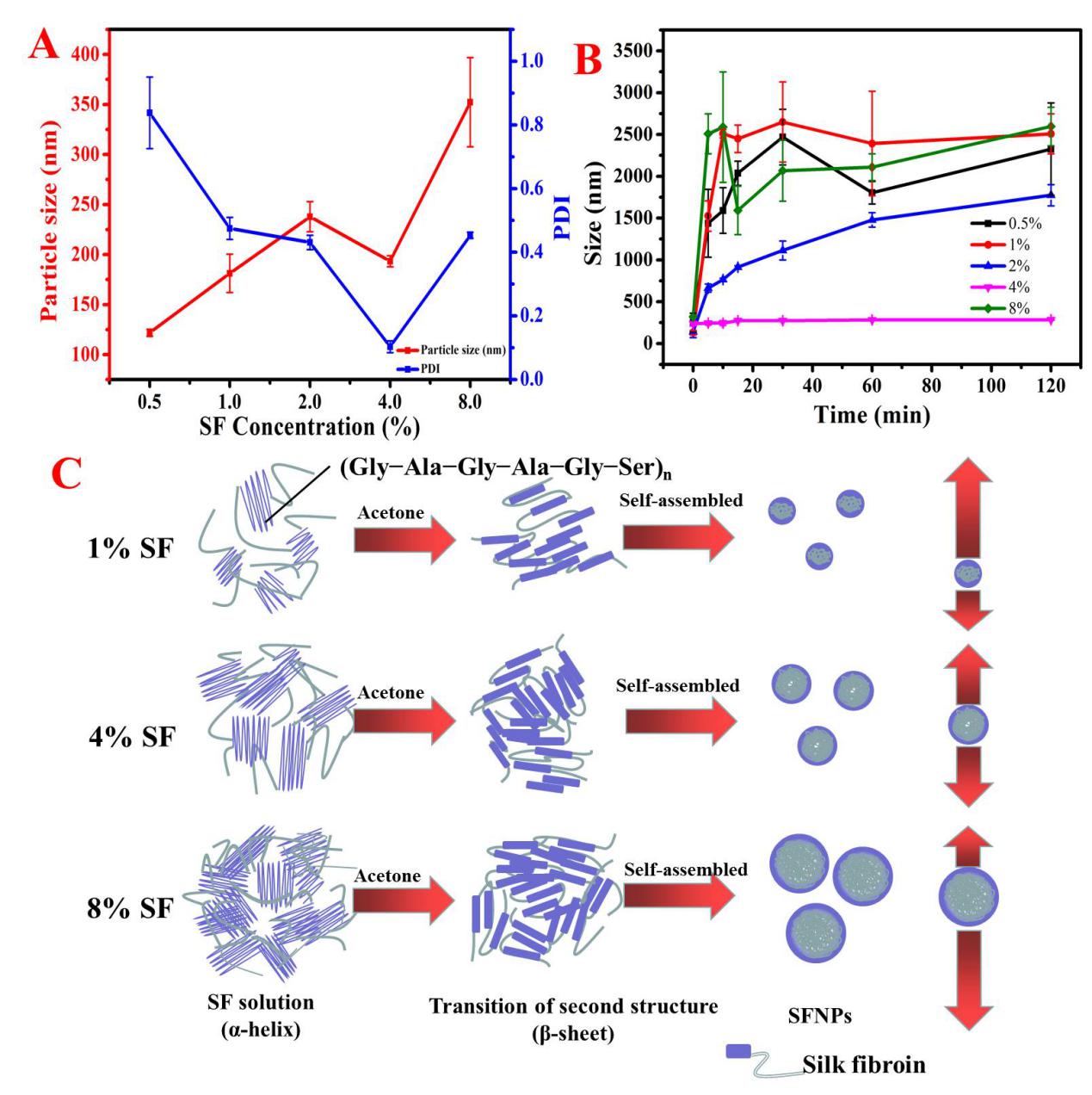


Fig S2 Formation and stability of SFNPs in pH7.4 PBS(10mM): (A) Particle size and PDI of SFNPs made by different concentration of SF, (B) Different SF concentration (0.5%, 1%, 2%, 4%, 8%) of preparation of SFNPs for 24h and stability of particle size in PBS at different time point, and (C)Principle of SFNPs formation at different SF concentration.


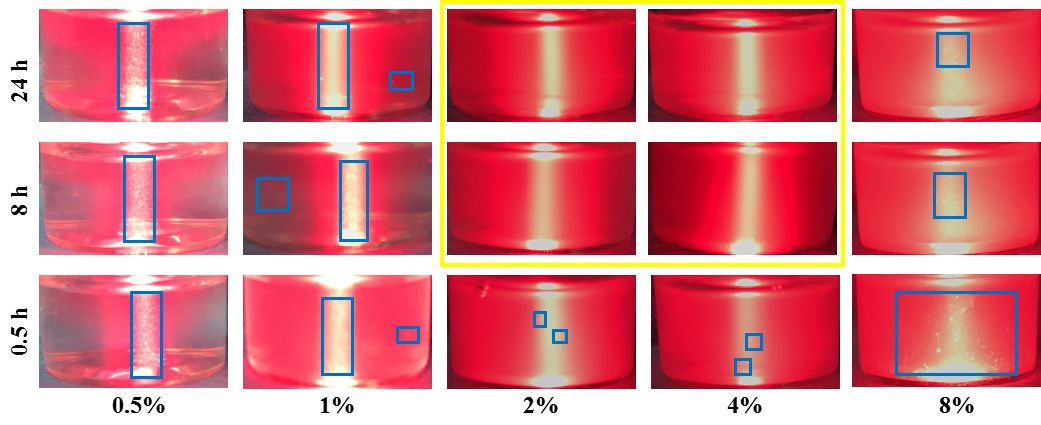


Fig S3 Different ways of preparation of SFNPs in the PBS (0.01M, pH=7.4) solution. The percentage below the picture (0.5%, 1%, 2%, 4%, 8%) represented the concentration of the pre-silk fibroin solution (SF solution) and the time on the left (0.5h, 8h, 24h) represented the acetone treatment time while SFNPs formation. Blue frame represented the large particles under the beam radiation.


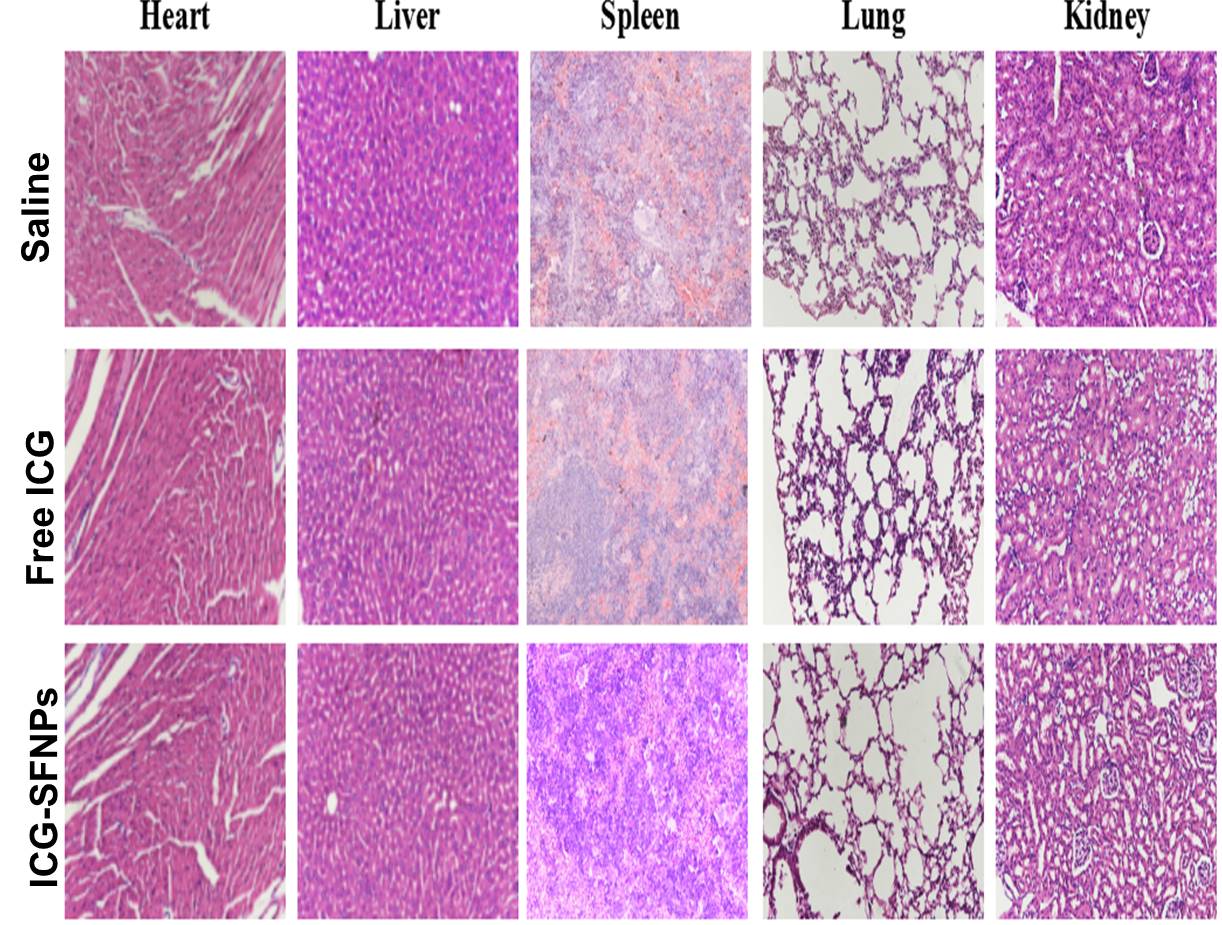


Fig S4 Hematoxylin and eosin (H&E) images of heart, liver, spleen, lung and kidney after treatment with different formulations.


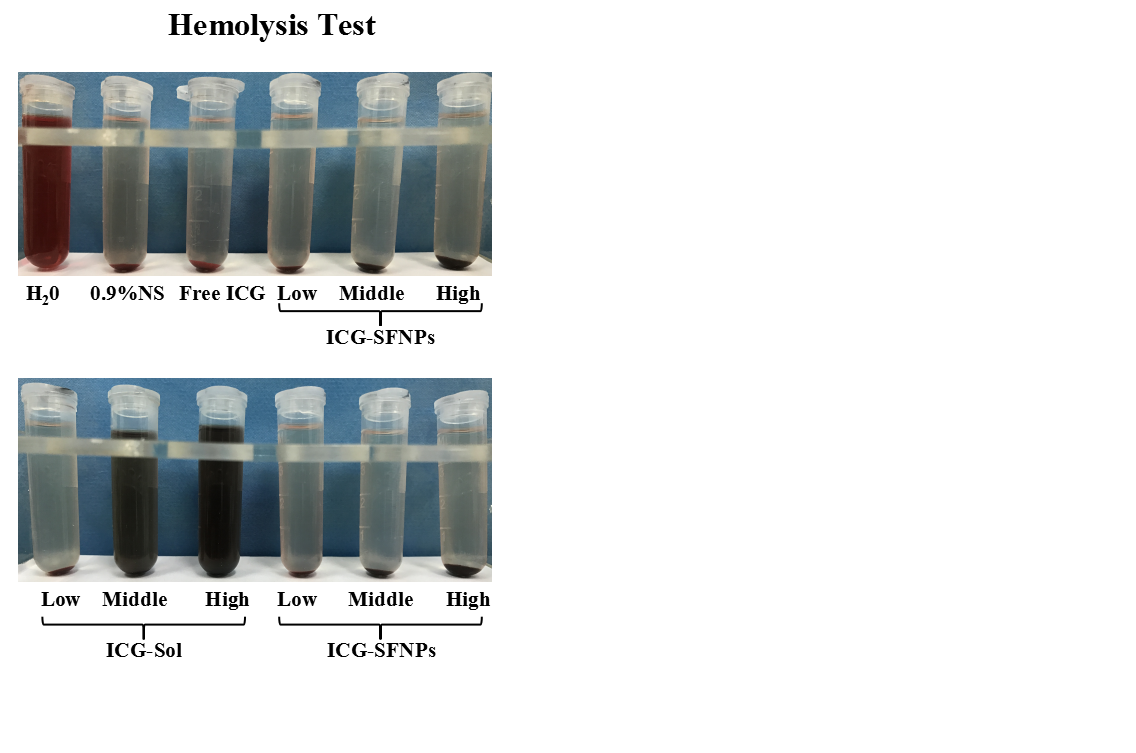


Fig S5 Hemolysis test of H2O, 0.9%NS, Free ICG and ICG-SFNPs at low, middle, high concentration.

**References**

1. Wang, H. Y.; Zhang, Y. Q., Processing silk hydrogel and its applications in biomedical materials. Biotechnol Prog 2015, 31 (3), 630-640.

2. Wenk, E.; Merkle, H. P.; Meinel, L., Silk fibroin as a vehicle for drug delivery applications. Journal of Controlled Release Official Journal of the Controlled Release Society 2011, 150 (2), 128-141.
